# Acute hepatitis a complicated by rhabdomyolysis in a young adult: a diagnostic challenge

**DOI:** 10.1093/omcr/omaf217

**Published:** 2025-10-29

**Authors:** Mohammed Albahloul Rajab, Abdalla Mohamed Hamed, Riad Elmabrouk esloughi, Amnah Meelad Ahmed, Nedal Mohamed Dhaw

**Affiliations:** Microbiology Department, Faculty of Medicine and Surgery, University of Zawia, Zawia City, Al Zawiya District, Western Libya State, P.O. Box 0023, Libya; Department of Microbiology, Faculty of Medicine and Surgery, University of Zawia, Zawia City, Al Zawiya District, Western Libya State, P.O. Box 0023, Libya; Department of Infectious Diseases, Tripoli Medical Centre, University of Tripoli Faculty of Medicine and Surgery, Tripoli City, Tripoli District, P.O. Box 0021, Libya; Department of Microbiology, Faculty of Medicine and Surgery, University of Zawia, Zawia City, Al Zawiya District, Western Libya State, P.O. Box 0023, Libya; Department of internal medicine, Faculty of Medicine and Surgery, University of Zawia, Zawia City, Al Zawiya District, Western Libya State, P.O. Box 0023, Libya

**Keywords:** hepatitis a, rhabdomyolysis, acute liver infection, case report, extra-hepatic manifestation

## Abstract

Hepatitis A is typically a self-limiting viral infection of the liver. Patients with hepatitis A may exhibit a wide range of clinical manifestations, from asymptomatic infection to fulminant liver failure. However, extra-hepatic manifestations such as rhabdomyolysis are exceedingly rare and, if unrecognized, may lead to delayed or missed diagnosis. We present the case of a 33-year-old Libyan female who developed rhabdomyolysis as a rare extra-hepatic complication of acute hepatitis A infection. The patient made a full recovery with timely conservative management. This case highlights the importance of maintaining clinical vigilance for atypical presentations of hepatitis A, as early recognition and supportive care are essential to prevent serious complications.

## Introduction

Hepatitis A is a non-enveloped RNA virus belonging to the Picornaviridae family and is a well-recognized cause of infectious hepatitis. It is primarily transmitted via the fecal-oral route. The incubation period typically ranges from 2 to 6 weeks, though higher doses of viral exposure may lead to a shorter incubation period [1]. Although hepatitis A virus primarily targets the liver, it can, in rare instances, lead to extrahepatic manifestations affecting other organs and systems. These manifestations are uncommon and occur in a minority of cases, with their severity and duration varying significantly among individuals [2]. Although rhabdomyolysis is an uncommon complication of acute non-fulminant hepatitis A, instances of rhabdomyolysis associated with acute renal failure have been documented [3].

## Case presentation

A 33-year-old Libyan female presented to our hospital with complaints of fever, fatigue, anorexia, and headache for three days prior to admission. The patient reported no notable medical, familial, or social history. She reported no nausea, vomiting, diarrhea, muscle weakness, or skin rash. Additionally, there was no history of recent travel, drug abuse, or contact with a known hepatitis A case. Additionally, the patient was not taking any prescribed or over-the-counter medications at the time of admission. On physical examination, the vital signs were within normal limits, the patient was conscious and afebrile. Abdominal examination showed no distension, and there was no evidence of hepatosplenomegaly, no tenderness upon palpation in all four quadrants, and normal bowel sounds. There were no signs of jaundice or scleral icterus. Examinations of the cardiac, central nervous, and respiratory systems were unremarkable. The patient was admitted for further evaluation and management. At admission, initial laboratory investigations showed elevated aspartate aminotransferase 114(U/l), alanine aminotransferase 232(U/l), and creatine phosphokinase levels 10 567 (U/l). There were no clinical or laboratory signs of liver insufficiency. The laboratory findings are summarized in Table 1. Bilirubin levels were normal, Urinalysis was positive for occult blood in the absence of red blood cells and demonstrated the presence of hyaline casts, suggestive of myoglobinuria. Two days into hospitalization, she developed diffuse, progressively worsening ascending myalgias accompanied by weakness. An abdominal ultrasound was performed and showed no acute pathology. Further investigations included a viral hepatitis panel (hepatitis B surface antigen, hepatitis C virus, hepatitis E virus, and HIV), all of which were negative. Anti-EBV IgM, anti-CMV IgM, and anti-HSV IgM were also not detected. Blood cultures demonstrated no significant bacterial growth. Autoimmune etiologies were not explored at that stage Table 1.

**Table 1:** Laboratory results of the patient during hospitalization.

Variable	Reference range (adults)	On admission	Day 4	Day 7
AST(U/l)	10–50	114	271	123
ALT(U/l)	20–70	232	413	140
CK(U/l)	55–170	10 567	11 344	8754
Urea (mg/dl)	7–20	9	12	10
Creatinine (mg/dl)	0.74–1.35	0.8	0.92	0.94
Bili-t (mg/dl)	0.3–1.2	0.6	0.5	0.6
BILI D (mg/dl)	0.0–0.3	0.2	0.1	0.1
GGT(IU/l)	5–40	21	10	17

AST: Aspartate transaminase, ALT: Alanine transaminase, CK, Billi-t: Bilirubin total, Bili-D: Bilirubin direct, GGT: Gamma glutamyl transferee.

In light of the unexplained clinical presentation, further testing was pursued with an IgM ELISA for hepatitis A virus (HAV), which subsequently returned positive for anti-HAV IgM. Due to logistical constraints and low clinical suspicion led to a delay in hepatitis A testing initially. Based on the comprehensive workup, the patient’s symptoms were attributed to rhabdomyolysis as a complication of acute hepatitis A infection. The patient was provided with supportive care, which included adequate fluid resuscitation, pain management, and careful monitoring of fluid balance, kidney function, and liver enzyme levels. After day 4 of admission, her condition steadily improved, and her liver function tests decreased. She was discharged after 7 days with advice for outpatient follow-up and a recommendation that close contacts receive the hepatitis A vaccine. The aspartate aminotransferase, alanine aminotransferase, and creatine phosphokinase levels became normal after 16 days.

## Discussion

Rhabdomyolysis occurring as a complication of acute non-fulminant hepatitis A is extremely rare, with only a limited number of case reports documenting this association in the literature [3, 4]. The pathogenesis of rhabdomyolysis in hepatitis A remains poorly understood. Proposed mechanisms include direct viral invasion of muscle tissue, immune-mediated myocyte injury, and systemic inflammatory responses resulting in muscle breakdown [5]. This case was diagnostically challenging due to the patient did not exhibit typical symptoms of hepatitis A, such as nausea, vomiting, or diarrhea. The typical clinical course of hepatitis A includes a prodromal symptoms such as malaise, joint pain (11%) and right upper quadrant pain, typically emerging weeks before the development of jaundice, which occurs in 40% to 70% of cases [6, 7]. Nevertheless, 14% of Acute HAV infection is usually silent or subclinical [8], which likely contributed to the delay in diagnosis in our patient. The eventual diagnosis of acute hepatitis A with rhabdomyolysis was confirmed only after positive anti-HAV IgM serology, highlighting an atypical and unexpected presentation. We observed increasing within ALT, AST and CK enzymes otherwise the laboratory investigation was within normal range [3]. in our case, the patient’s clinical presentation was initially perplexing, as her lack of a history of muscle pain or weakness made rhabdomyolysis an unlikely diagnosis at first. However, the markedly elevated creatine kinase (CK) levels, the development of ascending muscle pain and weakness, and the presence of hyaline cast in urine analysis strongly supported the diagnosis of rhabdomyolysis. Unfortunately, myoglobin levels were not available due to resource limitations. Although renal function remained stable in our patient, rhabdomyolysis carries a significant risk for acute kidney injury (AKI), necessitating early recognition and aggressive intravenous fluid therapy. Similar cases reported in the literature describe patients with acute hepatitis A presenting with muscle weakness, elevated transaminases, and increased CK levels [3, 4].

The primary management strategy for our patient focused on addressing rhabdomyolysis secondary to acute hepatitis A infection. Aggressive intravenous fluid resuscitation was initiated to prevent renal injury due to myoglobinuria. In this case, renal function remained stable, and the patient responded well to conservative management. However, in severe cases of rhabdomyolysis, particularly those complicated by hyperkalemia, oliguric renal failure, or persistent metabolic acidosis, the initiation of renal replacement therapy such as haemodialysis may become necessary [4].

This case highlights the importance of considering rhabdomyolysis as a potential extra-hepatic complication of acute hepatitis A infection, particularly in patients presenting with myalgia, muscle weakness, and elevated muscle enzymes. Early recognition and prompt initiation of supportive care, including aggressive hydration and electrolyte management, are essential to prevent serious complications such as acute kidney injury.
